# Investigation of bulk hybrid heterojunction solar cells based on Cu(In,Ga)Se_2_ nanocrystals

**DOI:** 10.1186/1556-276X-8-329

**Published:** 2013-07-19

**Authors:** Yu-Ting Yen, Yi-Kai Lin, Shu-Hao Chang, Hwen-Fen Hong, Hsing-Yu Tuan, Yu-Lun Chueh

**Affiliations:** 1 Department of Materials Science & Engineering, National Tsing Hua University, No. 101, Sec. 2, Kuang-Fu Rd., Hsinchu 30013, Taiwan; 2 Department of Chemical Engineering, National Tsing Hua University, No. 101, Sec. 2, Kuang-Fu Rd., Hsinchu 30013, Taiwan; 3 Institute of Nuclear Energy Research, No. 1000, Wenhua Rd., Jiaan Village, Longtan Township, Taoyuan County 32546, Taiwan; 4 Center For Nanotechnology, Material Science, and Microsystem, National Tsing Hua University, No. 101, Sec. 2, Kuang-Fu Rd., Hsinchu 30013, Taiwan

**Keywords:** Hybrid solar cell, Cu(In,Ga)Se_2_, Poly(3-hexylthiophene), Nanocrystal

## Abstract

This work presents the systematic studies of bulk hybrid heterojunction solar cells based on Cu(In, Ga)Se_2_ (CIGS) nanocrystals (NCs) embedded in poly(3-hexylthiophene) matrix. The CIGS NCs of approximately 17 nm in diameter were homogeneously blended with P3HT layer to form an active layer of a photovoltaic device. The blend ratios of CIGS NCs to P3HT, solvent effects on thin film morphologies, interface between P3HT/CIGS NCs and post-production annealing of devices were investigated, and the best performance of photovoltaic devices was measured under AM 1.5 simulated solar illumination (100 mW/cm^2^).

## Background

Photovoltaic (PV) devices, converting photon into electricity as an elegant and clean renewable energy, have attracted tremendous attentions on research and developments. Among emerging PV technologies, organic photovoltaic devices (OPV) composed of polymer matrices can be considered as promising third-generation solar cell due to its exceptional mechanical flexibility for versatile applications [1,2]. Moreover, the solution processes of OPV enables versatile and simple processes, including dip coating, ink jet printing, screen printing, and roll-to-roll method [3,4]. Nonetheless, OPVs suffer from the low carrier mobility issues, which hinder the performance far behind to conventional inorganic solar cells. In order to promote carrier mobility in OPV systems, inorganic semiconductor materials was introduced into OPV as electron acceptor materials, so called hybrid solar cells [5]. Hybrid solar cells utilize an advantage of intrinsically high carrier mobility from inorganic materials in organic matrices. By controlling of inorganic material into nanoscale, which can disclose the unique properties, such as enhanced absorption coefficient owing to quantum confinement [6], relatively high electron mobility, high surface area, and good thermal stability, providing alternative path for development of OPVs [7].

Typically, OPV composes of electron acceptors (e.g., [6,6]-phenyl-C61 butyric acid methyl ester (PCBM)) and hole transport conjugated polymers (e.g., poly(3-hexylthiophene (P3HT)) [8] as an active layer in the OPV. Owing to relative low carrier mobility and a similar band offset of most inorganic materials to PCBM. PCBM is usually replaced by inorganic nanomaterials as electron acceptor in most hybrid solar cells. Up to date, various inorganic semiconductors have been studied, including ZnO [9], TiO_2_[10], CdSe [11], CdS [12], PbSe [13], and PbS [14]. Among them, metal sulfides or selenides (i.e., Cd and Pb) were extensively investigated. Examples have been reported by as Alivisatos et al., indicating P3HT/CdSe nanorod hybrid solar cells achieve a remarkable power-conversion efficiency (PCE) of 1.7% [11]. Xu et al. have demonstrated a solar cell based on P3HT/PbSe NCs hybrids with a PCE of 0.13% [13]. However, Cd and Pb are considered as hazard elements to environments, which limit the hybrid solar cell systems as the commercialized product.

In this study, we report a hybrid solar cell based on CIGS NCs with a conjugated polymer P3HT as matrix. Chalcopyrite series material CIGS is well known as a direct bandgap material with an intrinsic high optical absorbing coefficient. Such superior characteristic and tunable optical energy gap engineering that matches well with the solar spectrum makes CIGS a promising PV material in the near future [15]. The blend ratios of CIGS NCs to P3HT, solvent effects on thin film morphologies, interface between P3HT/CIGS NCs and post-annealing of devices were investigated and the best performance of photovoltaic devices was measured. The approach combines non-toxic advantage of CIGS, benefitting a development in hybrid solar cells.

## Methods

### Synthesis of CIGS NCs

CIGS nanocrystals with stoichiometric of CuIn_0.5_Ga_0.5_Se_2_ was synthesized by chemical method. Oleylamine with 12 mL, 0.5 mmol of CuCl (0.0495 g), 0.25 mmol of InCl_3_ (0.0553 g), 0.25 mmol of GaCl_3_ (0.0440 g), and 1.0 mmol of elemental Se powder (0.0789 g) were mixed into a tri-neck beaker attached to the heating mantle. The beaker was purged by argon bubbling of oxygen and water at 130°C for 1 h. After purge, temperature was allowed to slowly increase to 265°C with slope of 2.3°C/min and held at 265°C for 1.5 h under vigorous stirring. The beaker was then cooled to room temperature by immersion into a cold water bath. The nanocrystals were extracted by a centrifugation process at 8,000 revolutions per minute (rpm) for 10 min by addition of 15 mL ethanol and 10 mL hexane. After two cycles of the centrifugation step, nanocrystals were precipitated and collected, while the supernatant was discarded. The extracted nanocrystals were re-dispersed in toluene or hexane for further device fabrication and characterization.

### Fabrication of photovoltaic device

Photovoltaic devices with a typical sandwich structure were fabricated, where the active layers are constructed using the CIGS NCs in combination with P3HT. Briefly, a 40-nm thick layer of filtered poly(3,4-ethylenedioxythiophene)-poly(styrenesulfonate) (PEDOT/PSS) was first spin-cast onto the indium tin oxide substrate with 400 rpm for 5 s and follow by 2,500 rpm for 40 s. Next, samples were dried at 120°C for 30 min under vacuum and transferred into a glove box filled by nitrogen gas. Then, an approximately 130-nm-thick P3HT/CIGS NC photoactive layer was deposited above the PEDOT/PSS layer by spin coating. The concentration of P3HT/CIGS NCs is 30 mg/mL using 1,2-dichlorobenzene as the solvent. The dried thin films were annealed at 120°C for 30 min. Finally, the Al electrodes (approximately 150 nm) were deposited by thermal evaporation, and through a shadow mask, resulted in a complete device with an active area of approximately 0.04 cm^2^.

### Measurements and characterizations

Powder X-ray diffraction (XRD) pattern was recorded on a Shimadzu 6000 X-ray diffractometer (Kyoto, Japan) with monochromated Cu-Kα irradiation (*λ* is approximately 0.154 nm). Morphology, microstructures, and atomic compositions of CIGS NPs were performed by field-emission scanning electron microscopy (JSE-6500F, JEOL, Akishima-shi, Japan) and high-resolution transmission electron microscopy (HRTEM, JEOL-3000F 300 kV) equipped with electron dispersive spectrometer. UV–vis absorption spectra were acquired using an optical spectrometer (Hitachi, U-4100, Minato-ku, Japan). Fourier transform infrared (FTIR) spectra were obtained by a Perkin Elmer Spectrum RXI spectrometer (Waltham, MA, USA). Photoluminescence (PL) spectra were measured under ambient conditions on a F-7000 spectrofluorometer (Hitachi) with an excitation at 400 nm. Current–voltage behaviors (Keithley 2410 source meter, Cleveland, OH, USA) were studied by adopting a solar simulator (San-Ei Electric, Osaka, Japan) with the AM 1.5 filter under an irradiation intensity of 100 W/cm^2^.

## Results and discussion

### Characterization of as synthesized CIGS NCs

**Figure 1:**
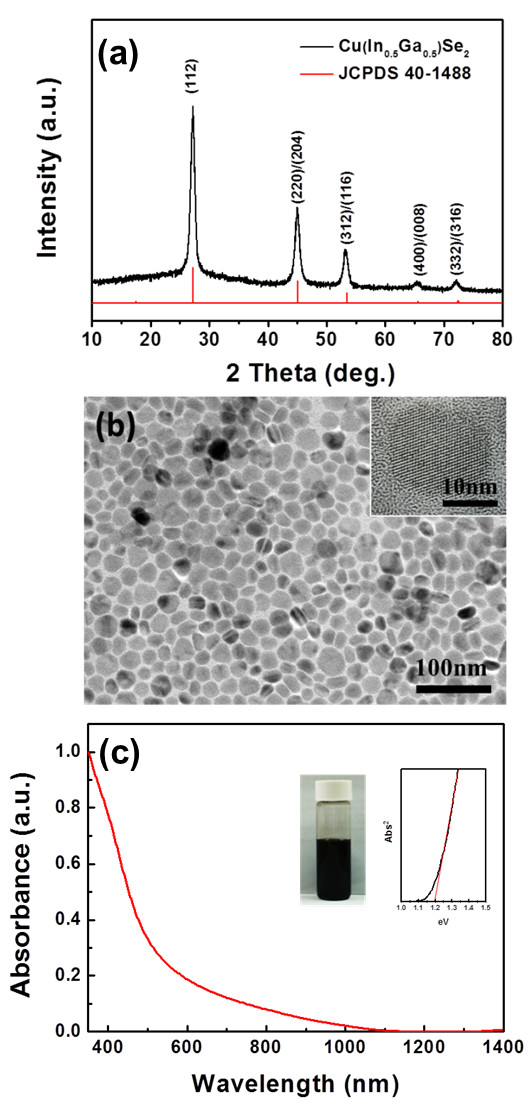
**XRD pattern (a), A TEM image (b), and UV-visible absorption spectra (c) of Cu(In_0.5_Ga_0.5_)Se_2_ NCs.** Inset in **(b)** shows the high-resolution TEM (HRTEM) images of Cu(In_0.5_Ga_0.5_^)^Se_2_ NCs. The NCs are calculated to be approximately 17 nm in average. Insets in **(c)** show the image of NCs dispersed in toluene solvent and the determination of band gap of approximately 1.2 eV by direct band gap method.

Figure 1a shows the XRD pattern of the as-synthesized CuIn_0.5_Ga_0.5_Se_2_ CIGS NCs. The peaks at approximates of 27°, 45°, 53°, 65°, and 72° were measured, which were consistent with the standard diffraction data of (112), (220)/(204), (312)/(116), (400)/(008), and (332)/(316) planes of Cu(In_0.5_Ga_0.5_)Se_2_ the chalcopyrite (JCPDS no. 40–1488), respectively. The size of nanocrystals can be calculated by the Scherrer equation *S* = *Kλ/*(*β*cos*θ*), where *K* is a constant (0.9), *λ* (1.54 Å) is the wavelength of the X-ray, *β* is the line broadening of full width at half the maximum (FWHM) intensity in radians, and *θ* is the Bragg angle. Therefore, the Bragg angle of (112) peak and the FWHM were applied to the Scherrer equation, the size of nanocrystals of approximately 18 nm can thus be extracted. The TEM image (Figure 1b) reveals that the average sizes of nanocrystals have a diameter of approximately 17 nm, which also match well with the size calculated from the XRD measurement. UV-visible absorption spectrum further investigated that the bandgap of CIGS NCs is approximately 1.2 eV; the black appearance shows its strong absorbance within a visible light window as shown in Figure 1c.

### Optical and compositional studies of CIGS NCs

**Figure 2:**
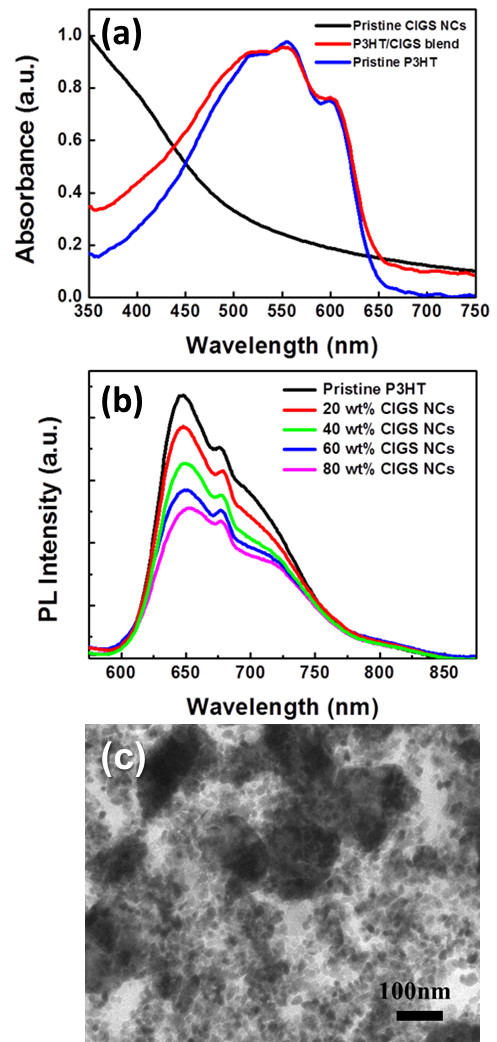
**Absorption spectra (a), photoluminescence spectra (*λ***_**exc**_** = 450 nm) (b), and TEM image (c).** Absorption spectra of the pristine P3HT, CIGS NCs, and P3HT:/CIGS NCs layer **(a)**, photoluminescence spectra (*λ*_exc_=450nm) of P3HT in composites, consisting of different concentration ratios between CIGS NCs and P3HT **(b)**, and TEM image of the CIGS NCs dispersed in P3HT matrix with the weight ratio of 60 wt.% **(c)**.

Optical studies of P3HT and P3HT/CIGS NC layer were characterized by absorption and PL spectroscopy. The pristine P3HT shows typical absorption spectra from 400 to 650 nm while the optical density in the P3HT/CIGS NC hybrid is simply the summation of the absorption spectra of the constituent parts (Figure 2a). Furthermore, no strong and distinct absorption peak was observed, indicating that there is a negligible ground-state charge-transfer between the polymer and the nanocrystals [16]. Figure 2b shows the PL spectra of P3HT/CIGS hybrid system with the excitation wavelength of 450 nm as a function of CIGS NC concentrations. Obviously, the PL intensity of the P3HT/CIGS NC hybrid decreases with the increase of CIGS NC concentrations compared to the pristine P3HT due to a non-radiative process. The decrease of PL spectra with CIGS NCs indicates a relatively effective energy transferred from the polymer to the CIGS NCs, resulting in the increasing of the non-radiative decay rate [17,18]. The non-radiative process was expected from the nanoscale interfaces between the P3HT and CIGS NCs, enabling excitons dissociated into free charges effectively, which can be confirmed by TEM image as shown in Figure 2c that the 60 wt.% CIGS NCs were dispersed quite uniformly in the P3HT matrix.

**Figure 3:**
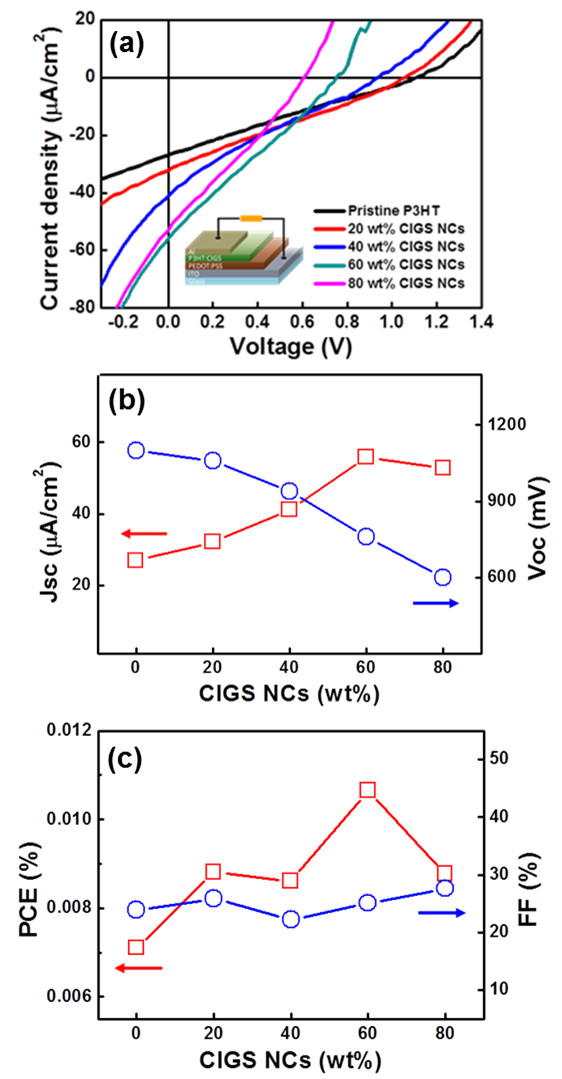
**I-V characteristics and Jsc, Voc, FF, and PCE of pristine and composition mixture of P3HT/CIGS NCs. (a)** I-V characteristics with the P3HT/CIGS NC composite layer at different mixing ratios and **(b,c)** Jsc, Voc, FF, and PCE as the function of the CIGS NCs concentrations.

Figure 3a shows the I-V characteristics with P3HT/CIGS NC composite layer at different mixing ratios. The short-circuit current (Jsc), opened circuit voltage (Voc), fill factor (FF), and PCE as the function of the CIGS NC concentrations were measured as shown in Table 1, respectively. In addition, the corresponding Jsc, Voc, FF, and PCE as the function of the CIGS NC concentrations were plotted as shown in Figure 3b,c, respectively. Obviously, as the concentration of CIGS NCs increases, the Jsc linearly increases due to the increasing of interfaces between P3HT and CIGS NCs, whereas the Voc decreases due to the decreasing of the shunt resistance. Consequently, the best photovoltaic devices with the optimal ratio (P3HT/CIGS NCs) of 60 wt.% can be found, with which the highest Jsc and Voc of approximately 59 μA/cm^2^ and approximately 0.76 V were measured, yielding the PCE (*η*) of approximately 0.011% with the FF of 0.25.

**Table 1:** Device measurement of P3HT/CIGS NC hybrid solar cells under AM 1.5 at different mixing ratios

**CIGS NCs (wt.%)**	**Jsc (μA/cm^2^)**	**Voc (mV)**	**FF (%)**	***η *(%)**
0	27	1,100	23.9	0.0071
20	32	1,060	25.9	0.0088
40	41	940	22.2	0.0086
60	59	760	25.1	0.0110
80	53	600	27.6	0.0080

### Solvent effects on CIGS NCs/P3HT hybrid solar cells

**Figure 4:**
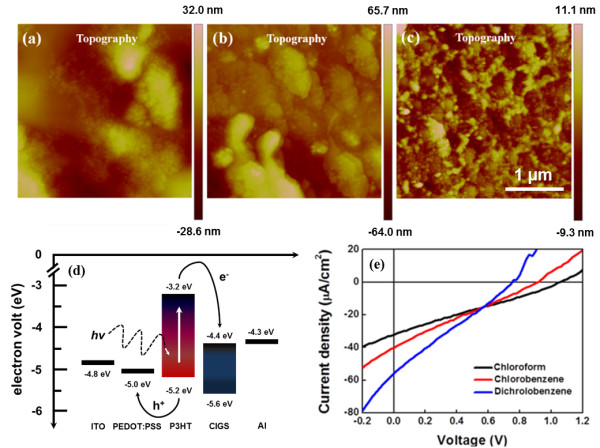
**AFM topography images (P3HT/CIGS films), energy diagram, and I-V characteristics (P3HT/CIGS hybrid solar cells).** AFM topography images of **(a)** choloform, **(b)** chlorobenzene, and **(c)** dichlorobenzene after spin-coating process. **(d)** Energy diagram of P3HT/CIGS hybrid solar cells and **(e)** its corresponding I-V characteristics.

By controlling the morphology of the active layer, the performance of the hybrid solar cell can be enhanced owing to the efficient charge transfer, transport, and collection strongly rely on the separated phases and morphologies in the polymer/NC layer [19]. The nanoscale morphology of an active layer mainly depends on the film preparation, including the use of different solvents, mixture of multiple solvents, control of solvent evaporation rate, and drying time [20]. Here, we investigated the morphology control in the P3HT/CIGS NC layer at different solvents, including chloroform, chlorobenzene, and dichlorobenzene as shown in Figure 4a,b,c, respectively. Comparing the atomic force microscope (AFM) images of chloroform, chlorobenzene, and dichlorobenzene-cast films, the dichlorobenzene-cast film achieves the smallest surface roughness of approximately 10 nm (approximately 25 to 30 nm for chloroform, approximately 40 to 50 nm for chlorobenzene). In order to compare the impact of the different morphologies and its corresponding device performance, all devices were fabricated in unity process except for the option of solvent adopted for spin coating of the active layer. Figure 4e shows a plot of the current density versus voltage for the three devices. Obviously, the Voc decreases from chloroform (1,060 mV), chlorobenzene (920 mV) to dichlorobenzene (760 mV) while the Jsc increases from chloroform (32 μA/cm^2^), chlorobenzene (40 μA/cm^2^) to dichlorobenzene (59 μA/cm^2^). As a result, the dichlorobenzene-based device exhibited the best PCE (0.011%), indicating high converting rate of photons to electrons. The enhanced PCE is due to the increase of the short-circuit current density, which can be attributed to an enhanced charge carrier transport for both charged carriers in the dichlorobenzene-cast active layer.

### Effects of interface treatment between CIGS NCs and P3HT

**Figure 5:**
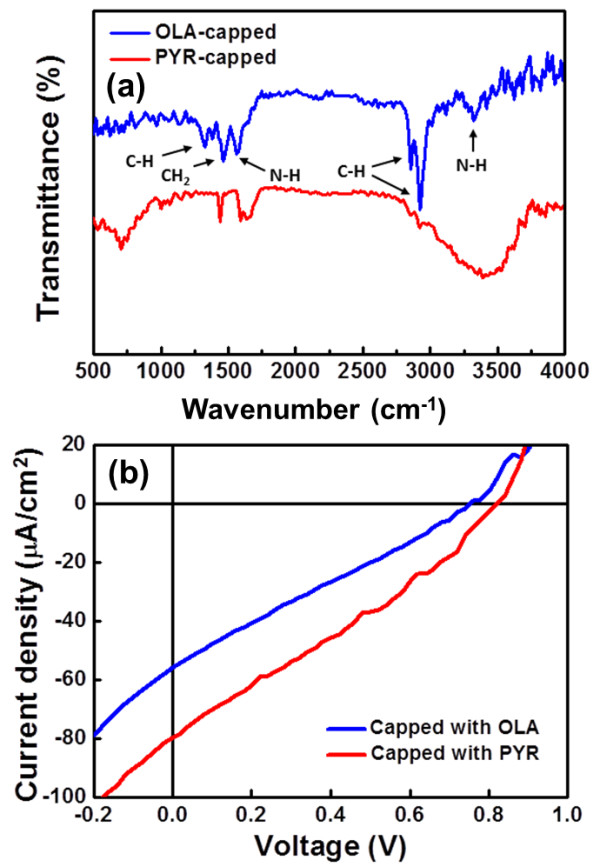
**FTIR of CIGS NCs (a) and I-V characteristics of photovoltaic devices (b) with and without pyridine treatment. (a)** CIGS NCs unrefluxed and refluxed by pyridine; **(b)** photovoltaic devices with and without pyridine treatment. (OLA, oleylamine; PYR, pyridine).

The crucial reason for the comparably poor performance of the hybrid solar cells might be due to carrier loss due to recombination on the surface of CIGS NCs. The surface of the as-synthesized CIGS NCs are end-capped with oleylamine as surfactant, which contains long alkyl chains with inherently dielectric properties, thus impeding a sufficient charge transport through the hybrid layer as well as charge separation at the interface between polymer/NCs [16]. Post treatment by pyridine-refluxed nanocrystals is a common way used for the reduction of interparticle distance thus enhancing the electrons/holes transported through the domain phases of nanocrystals [21]. Here, we employed the ligand exchange processes to substitute the oleylamine by the pyridine. A comparison of the FTIR transmission spectrum of the as-prepared and pyridine-treated CIGS NCs was characterized as shown in Figure 5a, and the corresponding I-V curves were measured as shown in Figure 5b for the hybrid solar cell before and after the pyridine treatment. Note that PV properties are highly related to the ligands capped onto surfaces of CIGS NCs. As a result, the Jsc increases after the pyridine treatment from 56 μA/cm^2^ to 69 μA/cm^2^ with the Voc of approximately 940 mV, yielding the enhanced power-conversion efficiency of approximately 0.017% with the fill factor of 0.26.The enhanced efficiency that pyridine-capped CIGS NCs enable more effective exciton dissociation at interfaces of P3HT/CIGS NCs compared with that of oleylamine-capped CIGS NCs.

### Effects of thermal treatments on CIGS NCs/P3HT hybrid solar cell

**Figure 6:**
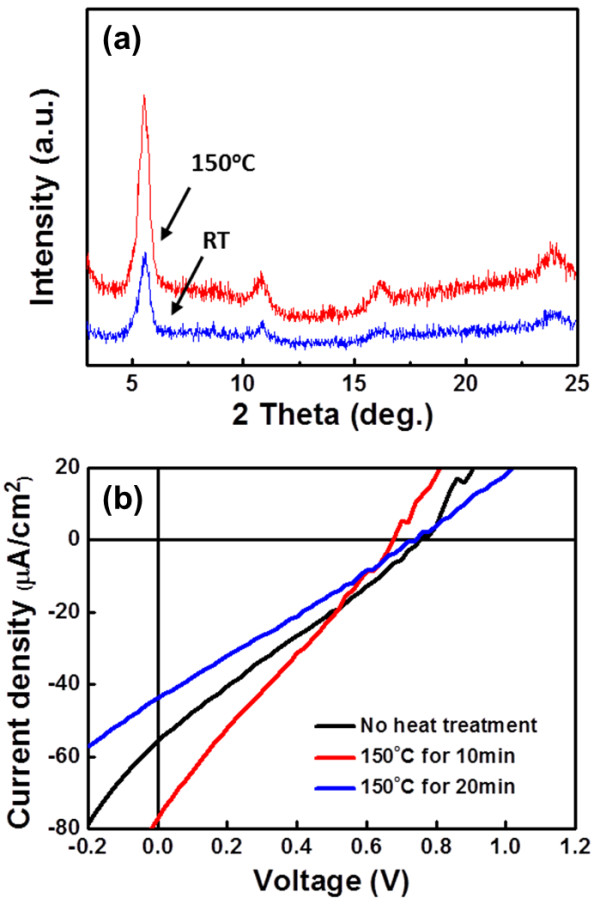
**XRD spectra (a) and I-V characteristics of P3HT/CIGS NC hybrid PV (b) with and without thermal annealing. (a)** devices with and without thermal annealing; **(b)** P3HT/CIGS NC hybrid PV at different annealing conditions.

The post-annealing is an effective way to enhance the performance of organic photovoltaic devices by enhancing nanoscale crystallinity so that an improved microstructure in the photoactive films can be achieved [22]. Here, the annealing was accomplished at 150°C for the hybrid solar cell after deposition of 100-nm-thick Al metal as electrode. The enhancement crystallinity of P3HT can be clearly observed from the XRD results as shown in Figure 6a, with which peaks with increased intensity at 6° and 24°, corresponding to interdigitated alkyl chains and interchain spacing in P3HT as a result of face-to-face packing from the thiophene rings can be observed. These two devices fabricated in the same manners were annealed at 150°C for 10 and 20 min, respectively. The former device exhibited the best PCE of 0.013% with the Jsc of 77 μA/cm^2^, while the PCE for the latter suddenly decreased, which may have resulted from the degradation of polymer.

## Conclusions

This work investigated and discussed on the bulk heterojunction of solar cell based on the P3HT/CIGS NC hybrid active layer. Approaches such as blend ratios of CIGS NCs, solvent effects on the morphologies, interface between P3HT/CIGS NCs, and device thermal treatments have been investigated to enhance the power-conversion efficiency of the hybrid solar cells in detail. The best performance of devices was fabricated from a blend ratio of 1 to 3 by weight in P3HT to CIGS NCs, dichlorobenzene as solvent, pyridine as surfactant, yielding the highest PCE of approximately 0.017%.

## Abbreviations

CIGS: Cu(In,Ga)Se_2_; NCs: Nanocrystals; P3HT: Poly(3-hexylthiophene); PV: Photovoltaic; OPV: Organic photovoltaic devices; PCBM: [6,6]-Phenyl-C61 butyric acid methyl ester; PCE: Power-conversion efficiency; PEDOT/PSS: Poly(3,4-ethylenedioxythiophene)-poly(styrenesulfonate); XRD: Powder X-ray diffraction; FTIR: Fourier transform infrared spectroscopy; HRTEM: High-resolution transmission electron microscopy; PL: Photoluminescence; FWHM: Full width at half maximum; Jsc: Short-circuit current; Voc: Opened circuit voltage; FF: Fill factor; OLA: Oleylamine; PYR: Pyridine.

## Competing interests

The authors declare that they have no competing interests.

## Authors’ contributions

YKL carried out the device fabrication and drafted the manuscript; SHC synthesized the CIGS nanocrystals; HFH provided useful solutions to the experimental issues and helped to revise the draft; HYT participated in the design of the study; YTY participated in the sequence alignment and helped to draft the manuscript; YLC carried out the TEM analysis, conceived the study, and organized the final version of the paper. All authors read and approved the final manuscript.
